# Haematological toxicity associated with antineoplastic drugs: a pharmacovigilance analysis based on the FDA adverse event reporting system database

**DOI:** 10.3389/fimmu.2026.1947521

**Published:** 2026-09-15

**Authors:** Xingnong Xu, Lei Ma

**Affiliations:** School of Pharmacy, East China University of Science and Technology, Shanghai, China

**Keywords:** antineoplastic drugs, FAERS, haematological toxicity, pharmacovigilance, time to onset

## Abstract

**Background:**

Antineoplastic drugs are effective for malignant tumours, but they frequently cause severe adverse drug reactions (ADRs), which seriously affect treatment continuity and patient prognosis. Post - marketing real - world safety monitoring is therefore critical.

**Methods:**

We conducted a retrospective pharmacovigilance study using FAERS (FDA Adverse Event Reporting System) data covering the period from 2004 Q1 to 2025 Q4. Safety signals were detected via disproportionate analysis, and associations were identified through LASSO and multivariable logistic regression, followed by an analysis of time to onset (TTO) for haematotoxicity.

**Results:**

A total of 347, 248 reports involving 367 antitumour drugs were analysed. Female patients (46.51%) outnumbered males (38.73%), with a median age of 59 years; patients aged 18 - 64.9 years represented 34.79% of the cohort. Fatal or life - threatening events were recorded in 78, 824 cases (22.70%). The leading drugs by report frequency were lenalidomide (ROR (Reporting Odds Ratio) = 1.51), methotrexate (ROR = 2.98), and rituximab (ROR = 3.69). Most events (61.65%) emerged within the first month, and median TTO differ significantly by sex. Furthermore, WSP analysis revealed that 26 of the top 30 drugs followed an early failure pattern.

**Conclusions:**

Our findings reveal a strong signal of haematological toxicity associated with antitumour agents, supporting enhanced routine monitoring to mitigate clinical risks. Nevertheless, confirmation through pharmacoepidemiological studies with rigorous causality assessment is required, owing to the inherent limitations of the FAERS spontaneous reporting system.

## 1 Introduction

Haematological toxicity, whose core manifestation is typically bone marrow suppression, is one of the most common and clinically significant adverse reactions associated with anticancer therapy; its specific manifestations include neutropenia, anaemia, and thrombocytopenia (1). Haematological toxicity represents an extremely frequent adverse event associated with cancer therapies. It may trigger severe complications including febrile neutropenia, which subsequently results in chemotherapy dose reductions, treatment interruptions, and even accelerated tumour progression. These outcomes exert detrimental impacts on patient prognosis, attenuate the antitumour activity of therapeutic agents, impair patients’ quality of life, and impose an additional economic and clinical burden on healthcare systems (2). Haematological toxicity is highly prevalent across a broad spectrum of anticancer therapies. In a systematic review and meta - analysis of breast cancer patients, the pooled prevalence of chemotherapy - induced myelosuppression was 47.9% (3). Among patients with colorectal cancer, the incidence of myelosuppression following first - line chemotherapy ranges from approximately 32.16% to 33.04% (4). Haematological toxicity is the most common dose - limiting toxicity in the treatment of lung and head and neck cancers, with an incidence exceeding 30% (5, 6). Chemotherapy represents the most well - recognized and clinically prevalent cause of myelosuppression. Chemotherapeutic agents, including platinum - based compounds, fluorouracil derivatives and taxanes, exert non - selective cytotoxic effects on rapidly proliferating haematopoietic progenitor cells (7). For instance, carboplatin and gemcitabine have been demonstrated to modify individual susceptibility to myelosuppression through their impact on enhancer mutations (8). Targeted agents are likewise capable of triggering prominent haematological adverse events. For example, imatinib can elicit myelosuppression in patients with gastrointestinal stromal tumours (GIST), and the incidence of such toxicities varies across ethnic populations (9). Although regimens based on immune checkpoint inhibitors generally exhibit acceptable overall safety, haematological toxicity remains a common adverse event. The incidence of myelosuppression was 51.7% when nivolumab was combined with chemotherapy and immunotherapy as first - line treatment for squamous cell carcinoma of the oesophagus (10).

Currently, growth factor support constitutes the primary therapeutic approach for myelosuppression; however, its clinical efficacy remains suboptimal. Granulocyte colony - stimulating factor (G - CSF) is chiefly used to manage neutropenia, erythropoiesis - stimulating agents (ESAs) for anaemia, and platelet transfusions for thrombocytopenia; nevertheless, none of these interventions can provide concurrent protection across multiple haematopoietic lineages (11). In a real - world cohort of patients diagnosed with extensive - stage small cell lung cancer, analysis of 1239 eligible cases revealed that 98.6% of the study population experienced at least one myelosuppression event throughout chemotherapy. Notably, among all patients with myelosuppression, 62.1% suffered from suppression affecting ≥ 3 haematopoietic lineages, and only 33.9% had suppression confined to 1–2 lineages. This finding underscores the critical limitation of current single - lineage supportive strategies, which cannot adequately address the clinical scenario of multi - lineage suppression (12). At present, much research into the haematological toxicity of chemotherapy and targeted therapies still relies on clinical trial data, while the collection and analysis of real - world data remain insufficient. In an analysis of the impact of bariatric surgery on oral anticancer drugs, the study was based solely on 571 patients from a single cancer centre, and the generalisability of its conclusions requires validation by larger, multicentre real - world data (13). The swift evolution of immunotherapies, targeted agents, and combination therapies presents novel challenges to pharmacovigilance. Real - world evidence indicates that some new anticancer drugs are linked to serious haematological adverse events, such as thrombotic microangiopathy (TMA), with a substantially rising number of reported cases over the last ten years. However, post - marketing safety data for these agents often trail their actual use, and many suspected safety signals await verification through additional confirmatory research (14). Cancer patients commonly undergo polypharmacy, and intricate drug interactions are easily underestimated or ignored during pharmacovigilance analyses. Broader real - world datasets are therefore urgently required to improve the current evaluation framework. While existing pharmacokinetic mathematical models have been established to predict haematological toxicity risks, their predictive accuracy remains unsatisfactory, limiting large - scale clinical application (15).

As one of the world’s largest spontaneous adverse event reporting systems, the FAERS database serves as a cornerstone of real - world drug safety data. It captures rare, delayed, or population - specific adverse events that are difficult to detect in clinical trials due to limited sample sizes, short follow - up periods, or highly selected patient populations, thereby demonstrating its unique value in identifying unknown or unexpected safety signals (16, 17). Furthermore, FAERS represents a core platform for pharmacovigilance signal detection and quantitative analysis. Utilizing the large - scale adverse event dataset contained in the database, researchers can calculate the strength of association between specific drugs and target adverse events via classical metrics such as the reporting odds ratio (ROR). This method effectively identifies adverse reactions whose observed frequency exceeds the expected random background level, so as to efficiently screen out risk signals that require priority intervention (18, 19). Leveraging FAERS data, one can generate a thorough overview of drug - associated adverse events, including the involved System Organ Classes (SOCs), leading Preferred Terms (PTs), patient demographics (e.g., age and sex), and chronological patterns of event occurrence. Such a multifaceted assessment provides clinicians with safety insights that are more granular and comprehensive than those found in conventional package inserts (20, 21). As a core resource for drug safety monitoring, the FAERS database plays a vital role in identifying unknown drug-related risks, quantifying association magnitudes, and delineating drug safety profiles. Its strengths stem from its real - world setting, large - scale data resources, and standardised analytical framework (22). FAERS is a publicly accessible database widely used for the analysis of adverse drug reactions. Considering the clinical benefits to patients, a comprehensive analysis is required to explore the relationship between anticancer drugs and haematological toxicity, as well as the factors influencing this relationship.

Accordingly, this retrospective study applied disproportionality analysis and regression models to investigate the safety signals of anticancer drugs using FAERS data. We further analysed the influencing factors of the corresponding adverse reactions and conducted subgroup stratification analysis. The results of this study aim to provide real - world evidence supporting rational medication and safety management of antineoplastic agents in clinical oncology.

## 2 Methods

### 2.1 Data sources

This study involved mining and analysing data on anticancer drugs from the FAERS database, covering the period from the first quarter of 2004 to the fourth quarter of 2025. The ASCII (American Standard Code for Information Interchange) data files extracted from the FAERS database comprise seven subsets: patient demographic and administrative information (DEMO), drug management information (DRUG), report source records (RPRS), adverse reaction codes (REAC), drug therapy records (THER), outcome data (OUTC), and drug indication records (INDI). These datasets were linked using the primaryid field as the primary key, which uniquely identifies each FAERS report and is common across all seven data files.

### 2.2 Data processing

Consistent with FDA guidelines for duplicate data removal, all duplicate entries were eliminated. For records sharing the same medical record number, the latest version was preserved, and record linkage was established using primary identification variables. In other words, for records with the same primaryid, the most recent version will be retained based on the FDA_DT field (the date the FDA received the case). Primary suspect (PS) drugs were identified by retrieving cases mapped to the standardized Preferred Term (PT) “Haematological toxicity” defined by the Medical Dictionary for Regulatory Activities (MedDRA 28.1). In cases where multiple drugs were reported with a PS designation within the same primaryid (i.e., combination therapy regimens), all PS - designated drugs were retained for analysis as separate drug - event pairs. MedDRA term search strategy and their encoding hierarchy are shown in **Supplementary Table S1**. All extracted drug names were further normalized using the Anatomical Therapeutic Chemical (ATC) classification system via the official publicly accessible database (https://atcddd.fhi.no/atc_ddd_index/). **Figure 1** systematically illustrates the full workflow for extraction, deduplication and standardization of adverse events related to haematological toxicity and anticancer agents.

**Figure 1:**
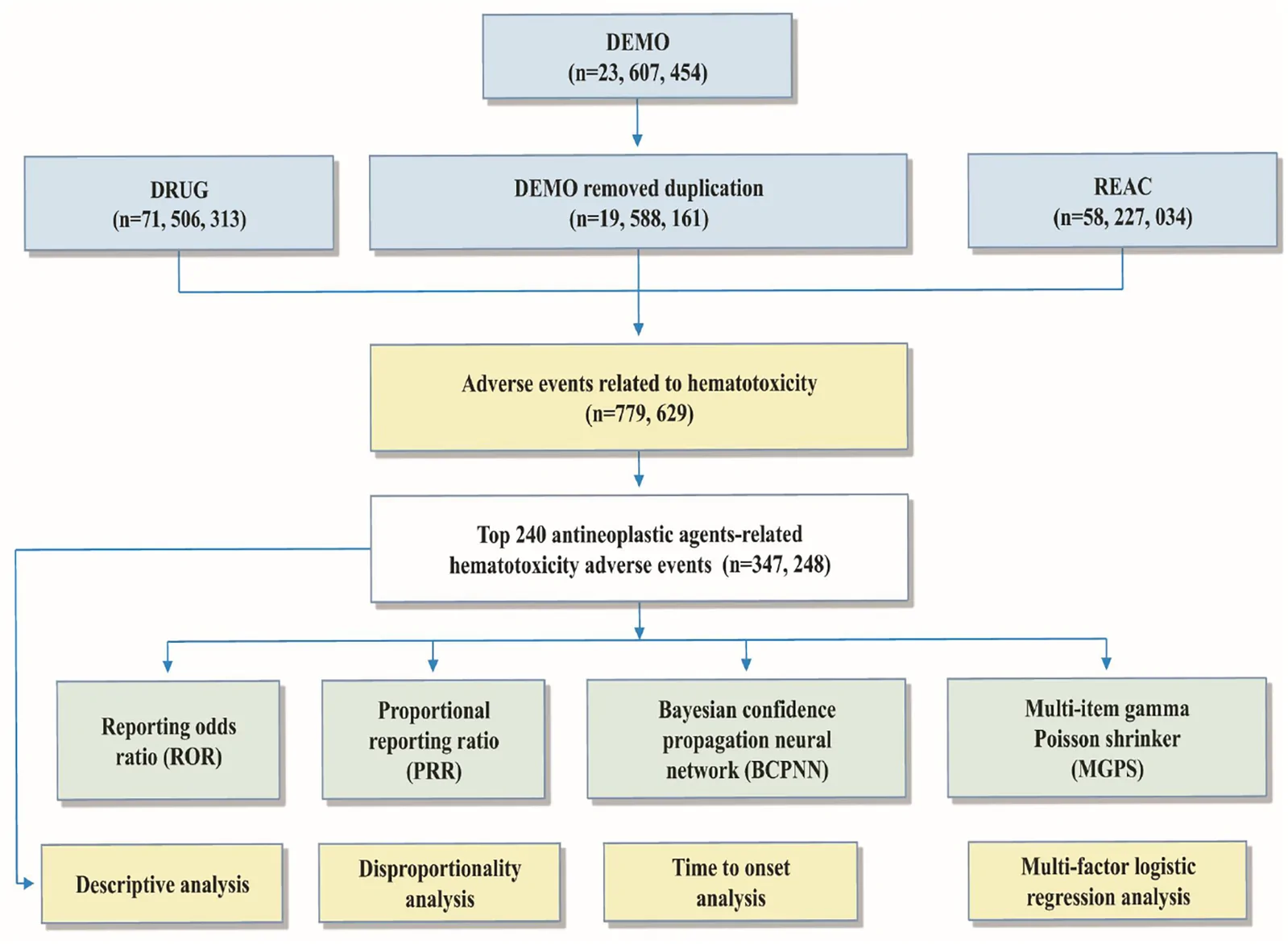
Flow diagram depicting the process of data extraction and cleaning.

### 2.3 Regression analysis

Suspected drugs were initially screened by univariate analysis using three inclusion criteria: reporting odds ratio (ROR) with a 95% confidence interval (CI) lower limit > 5, event frequency > 1000, and adjusted p - value < 0.01 (23–25). Those that achieved statistical significance (p < 0.01) were further evaluated via least absolute shrinkage and selection operator (LASSO) regression. A multivariate logistic model was then built with LASSO - selected drug variables and baseline patient characteristics as independent variables, aiming to determine specific statistical signal for haematotoxicity associated with antitumour drug therapy. The logistic regression was constructed using a case and non - case design, which is the standard approach for spontaneous reporting system analyses.

### 2.4 Time to onset analysis

Time to onset (TTO) is defined as the interval between the date of drug initiation (START_DT in the THER file) and the date of the adverse event (EVENT_DT in the DEMO file). Dates that were inaccurate, missing, or incorrectly entered were excluded from the analysis. In addition, the TTO data were analysed using the median, the first quartile (IQR), and the Weibull shape parameter (WSP). The Weibull distribution is defined by a scale parameter (α) and a shape parameter (β). Three patterns of ADE failure modes were determined based on β values and their 95% CIs. Early failure occurs when β < 1 (95% CI < 1), corresponding to a decreasing temporal trend in ADE risk. Random failure is identified when β approximates 1 (95% CI includes 1), indicating stable ADE risk over time. Wear - out failure is defined as β > 1 (95% CI > 1), representing a progressively rising ADE risk over time. To evaluate the potential for selection bias introduced by the exclusion of reports with missing or invalid dates, we conducted a systematic comparison between the TTO - evaluable cohort (reports with complete and valid onset dates) and the TTO - excluded cohort (reports with missing or invalid dates). Reports in which the EVENT_DT and START_DT were identical (i.e., TTO = 0 days) were retained in the analysis and assigned a TTO of 0.5 days.

### 2.5 Statistical analysis

Four established pharmacovigilance algorithms were used for adverse reaction signal analysis: the Reporting Odds Ratio (ROR), Proportional Reporting Ratio (PRR), Bayesian Confidence Propagation Neural Network (BCPNN), and Multi - item Gamma Poisson Shrinker (MGPS). The ROR was selected as the primary statistical indicator, and a higher ROR value suggests a stronger association between the antineoplastic drug and the risk of haematological toxicity. The detailed calculation formulas for these methods are provided in **Tables 1**, **2** (23–25). Consistent with pharmacovigilance best practices, all four algorithm outputs - including point estimates and their respective 95% confidence intervals or lower credibility limits - are reported simultaneously in the results section. Specifically, we present the ROR with 95% CI, the PRR with χ² statistic, the EBGM (Empirical Bayes Geometric Mean) with its 5th percentile (EBGM05), and the IC (Information Component) with its 5th percentile (IC025). Signals were considered statistically detectable only when all four algorithms concurrently met their respective threshold criteria, thereby reducing false-positive risk. All statistical analyses were performed using R software (version 4.3.1); details regarding the specific packages used and the analytical procedures are provided in **Supplementary Code S1**.

**Table 1:** Two - by - two contingency table applied in disproportionality analysis.

Type of drug	N of target adverse events	N of other adverse events	Total
Target drug	a	b	a+b
All other drugs	c	d	c+d
Total	a+c	b+d	a+b+c+d

**Table 2:** Summary of primary signal detection algorithms adopted in this study.

Algorithm	Publicity	Continuity correction	Standard for generating signals
ROR	$ROR\;=\;\frac{a/b}{c/d}$	+0.5 to all cells if any cell = 0	Lower 95% CI > 1, N ≥ 3
PRR	$PRR\;=\;\frac{\text{a}/\left(\text{a}+\text{b}\right)}{c/\left(c+d\right)}$	+0.5 to all cells if any cell = 0	χ² ≥ 4, PRR ≥ 2, N ≥ 3
	χ² = [(ad-bc)^2](a+b+c+d)/[(a+b)(c+d)]		
MGPS	$EBGM\;=\;\frac{\text{a}\left(\text{a}+\text{b}+\text{c}+\text{d}\right)}{\left(a+b\right)\left(a+c\right)}$ 95% CI = eln(EBGM) ± 1.96(1/a+1/b+1/c+1/d) ^ 0.5	Empirical Bayesian shrinkage (no artificial correction needed)	EBGM05 > 2
BCPNN	$IC\;=\;\log_{2}\;\frac{a\left(a+b+c+d\right)}{\left(a+b\right)\left(a+c\right)}$ 95% CI = E (IC) ± 2V (IC)^0.5	Empirical Bayesian shrinkage (no artificial correction needed)	IC025 > 0, N > 0

CI, confidence interval; EBGM, Empirical Bayes Geometric Mean; EBGM05, the lower 5th percentile of EBGM; IC, Information Component; IC025, the lower 5th percentile of IC; N, number of reports. A continuity correction of 0.5 was applied to zero - cell cases for frequentist algorithms (ROR and PRR). For Bayesian algorithms (MGPS and BCPNN), shrinkage estimation inherently stabilises variance without cell - wise correction.

## 3 Results

### 3.1 Descriptive analysis

Data mining revealed that 367 anticancer drugs were positively correlated with haematological toxicity, a finding supported by 347, 248 case reports (**Table 3**). The majority of anticancer drug - related haematological adverse events (AEs) originated from the United States (115, 642; 33.30%) and Japan (32, 148; 9.26%). After excluding patients with missing age data (n = 97, 142; 27.97%), the median age was 59 years (IQR: 34.8 - 74), with the largest proportion of participants (120, 799; 34.79%) concentrated in the 18 - 64.9 age group. The majority of patients had body weight within the 50–100 kg range (n = 95, 154; 27.40%). Among cases with documented gender (excluding 51, 252 unrecorded; 14.76%), females (161, 501; 46.51%) outnumbered males (134, 495; 38.73%) by a notable margin. Reporting sources were predominantly physicians (151, 397; 43.60%) and health professionals (54, 566; 15.71%). Fatal or life - threatening outcomes occurred in 78, 824 patients (22.70%). As depicted in **Figure 2**, the annual report count for haematological adverse events related to anticancer drugs has shown a steady increase since 2004, reaching a peak in 2024 with 32, 873 cases (9.47% of the total 21 - year cohort).

**Table 3:** Clinical characteristics of haematological toxicity reports.

Characteristics	Case number (n)	Case proportion (%)
Gender		
Female	161, 501	46.51%
Male	134, 495	38.73%
missing	51, 252	14.76%
Weight		
<50 kg	17, 229	4.96%
>100 kg	7, 763	2.24%
50∼100 kg	95, 154	27.40%
missing	227, 102	65.40%
Age		
<18	19, 231	5.54%
>85	4, 341	1.25%
18∼64.9	120, 799	34.79%
65∼85	105, 735	30.45%
missing	97, 142	27.97%
OCCP_COD		
Medical Doctor	151, 397	43.60%
Health professional	54, 566	15.71%
Consumer	51, 390	14.80%
Other	50, 075	14.42%
Pharmacist	22, 050	6.35%
missing	16, 770	4.83%
Reporting country		
United States	115, 642	33.30%
Japan	32, 148	9.26%
China	26, 432	7.61%
France	24, 210	6.97%
Germany	18, 288	5.27%
Canada	16, 880	4.86%
Outcomes		
Congenital Anomaly	89	0.03%
Death	54, 840	15.79%
Disability	1, 712	0.49%
Hospitalization	111, 654	32.15%
Life-Threatening	23, 984	6.91%
Required Intervention	568	0.16%
Other	134, 712	38.79%
missing	19, 689	5.67%

**Figure 2:**
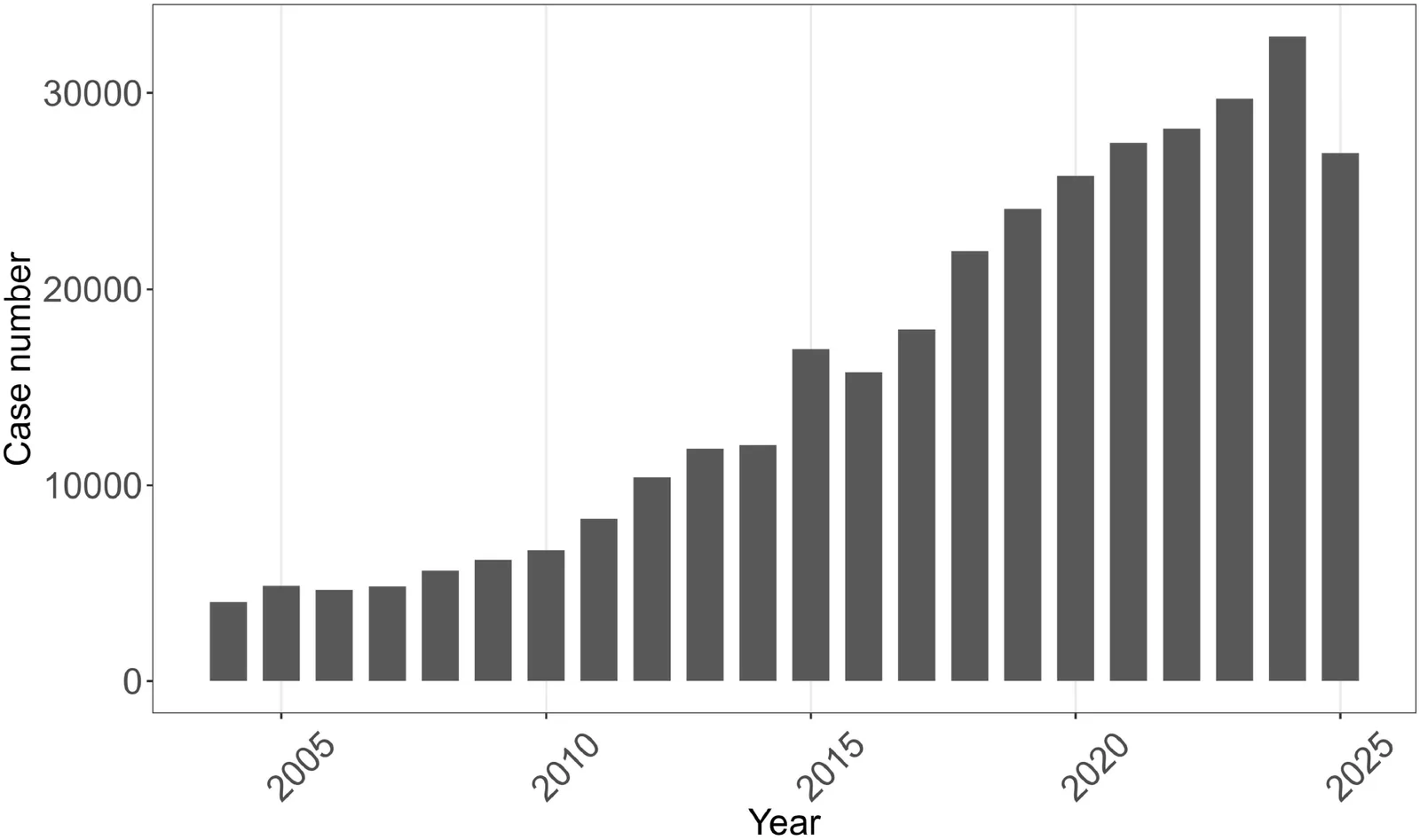
Number of reports per year.

### 3.2 Disproportionality analysis

Following data preprocessing and analysis, 367 anticancer drugs were found to present positive haematotoxicity signals. The top 30 drugs ranked by report count are listed in **Table 4**, which simultaneously reports ROR with 95% CI, PRR with χ² statistic, EBGM with EBGM05, and IC with IC025. The top five medications with prominent haematotoxicity signals included lenalidomide, methotrexate, rituximab, carboplatin, and palbociclib, with ROR values of 1.51 (95% CI: 1.49 - 1.51), 2.98 (95% CI: 2.93 - 3.04), 3.69 (95% CI: 3.63 - 3.76), 7.46 (95% CI: 7.31 - 7.62), and 3.08 (95% CI: 3.01 - 3.15), respectively. The largest magnitude observed for cytarabine (ROR = 14.82, 95% CI: 14.29 - 15.36; PRR = 9.58, χ² = 37, 808.34; EBGM = 9.53, EBGM05 = 9.19; IC = 3.25, IC025 = 3.20) and the complete matrix of all four signal metrics for all 367 drugs is provided in **Supplementary Table S2**. Subsequent stratified categorization analysis of all included antineoplastic agents demonstrated that cytotoxic agents contributed the largest number of haematological toxicity cases and exhibited the highest ROR value among all drug categories. Molecularly targeted therapies accounted for the second - highest case volume, while antibody - drug conjugates ranked second in terms of the magnitude of ROR values (**Figure 3**). Furthermore, among the 367 anticancer drugs included in this analysis, the official package inserts of 348 contained explicit warnings related to haematological toxicity risk, whereas the remaining 19 drugs did not include such safety warnings, with representative examples including toremifene, goserelin and exemestane.

**Table 4:** Top 30 antineoplastics implicated in haematological adverse reactions.

Ranking	Drug	Cases	ROR (95% CI)	PRR (χ²)	EBGM (EBGM05)	IC (IC025)
1	Lenalidomide^*^	21246	1.51 (1.49-1.53)	1.48 (3331)	1.47 (1.44)	0.55 (0.53)
2	Methotrexate^*^	14837	2.98 (2.93-3.04)	2.77 (17114.68)	2.73 (2.69)	1.45 (1.43)
3	Rituximab	14439	3.69 (3.63-3.76)	3.34 (24210.50)	3.30 (3.24)	1.72 (1.70)
4	Carboplatin^*^	11119	7.46 (7.31-7.62)	5.95 (47014.76)	5.88 (5.76)	2.56 (2.52)
5	Palbociclib^*^	8936	3.08 (3.01-3.15)	2.85 (11026.35)	2.83 (2.76)	1.50 (1.47)
6	Doxorubicin^*^	8528	8.13 (7.93-8.33)	6.34 (39543.31)	6.29 (6.13)	2.65 (2.62)
7	Bevacizumab	8390	3.17 (3.09-3.24)	2.92 (10882.87)	2.90 (2.83)	1.53 (1.50)
8	Ruxolitinib^*^	8108	3.46 (3.38-3.54)	3.15 (12270.23)	3.13 (3.06)	1.65 (1.61)
9	Gemcitabine^*^	7502	9.02 (8.79-9.27)	6.85 (38681.14)	6.80 (6.62)	2.76 (2.73)
10	Venetoclax^*^	7351	4.94 (4.82-5.06)	4.27 (19014.82)	4.24 (4.14)	2.08 (2.05)
11	Cyclophosphamide^*^	7342	7.39 (7.20-7.58)	5.90 (30823.86)	5.85 (5.70)	2.55 (2.51)
12	Pembrolizumab	5562	2.70 (2.62-2.77)	2.53 (5307.30)	2.52 (2.45)	1.33 (1.29)
13	Paclitaxel^*^	5554	4.19 (4.07-4.31)	3.72 (11414.05)	3.70 (3.59)	1.89 (1.84)
14	Oxaliplatin^*^	5481	4.68 (4.54-4.81)	4.08 (13185.56)	4.06 (3.94)	2.02 (1.98)
15	Ibrutinib^*^	5370	2.06 (2.01-2.12)	1.98 (2694.13)	1.97 (1.92)	0.98 (0.94)
16	Imatinib^*^	5149	2.37 (2.30-2.44)	2.25 (3678.95)	2.24 (2.17)	1.16 (1.12)
17	Cisplatin^*^	5101	8.77 (8.49-9.06)	6.71 (25632.09)	6.67 (6.46)	2.74 (2.69)
18	Trastuzumab	4982	3.96 (3.84-4.08)	3.54 (9412.33)	3.53 (3.42)	1.82 (1.77)
19	Capecitabine^*^	4809	2.18 (2.12-2.25)	2.09 (2810.86)	2.08 (2.02)	1.06 (1.01)
20	Cytarabine^*^	4754	14.82 (14.29-15.36)	9.58 (37808.39)	9.53 (9.19)	3.25 (3.20)
21	Docetaxel^*^	4727	2.41 (2.34-2.48)	2.28 (3527.78)	2.28 (2.21)	1.19 (1.14)
22	Peginterferon Alfa 2A^*^	4576	4.12 (3.99-4.25)	3.67 (9195.13)	3.65 (3.54)	1.87 (1.82)
23	Azacitidine^*^	4495	8.60 (8.31-8.90)	6.61 (22158.43)	6.58 (6.36)	2.72 (2.67)
24	Fluorouracil^*^	4427	6.55 (6.33-6.77)	5.37 (16289.21)	5.34 (5.17)	2.42 (2.37)
25	Pomalidomide^*^	4358	1.30 (1.26-1.34)	1.28 (284.39)	1.28 (1.24)	0.36 (0.31)
26	Nivolumab	4232	1.65 (1.60-1.70)	1.61 (1007.46)	1.60 (1.56)	0.68 (0.64)
27	Bortezomib^*^	3848	3.12 (3.02-3.23)	2.88 (4892.40)	2.87 (2.78)	1.52 (1.47)
28	Letrozole	3835	5.38 (5.20-5.57)	4.58 (11138.16)	4.57 (4.41)	2.19 (2.14)
29	Etoposide^*^	3480	9.19 (8.83-9.55)	6.94 (18326.34)	6.91 (6.64)	2.79 (2.73)
30	Sunitinib^*^	3346	2.39 (2.31-2.48)	2.27 (2453.14)	2.26 (2.18)	1.18 (1.12)

ADE, adverse drug event; CI, confidence interval; ROR, Reporting Odds Ratio; PRR, Proportional Reporting Ratio; BCPNN, Bayesian Confidence Propagation Neural Network; MGPS, Multi - item Gamma Poisson Shrinker; ^*^warnings related to haematological toxicity risk.

**Figure 3:**
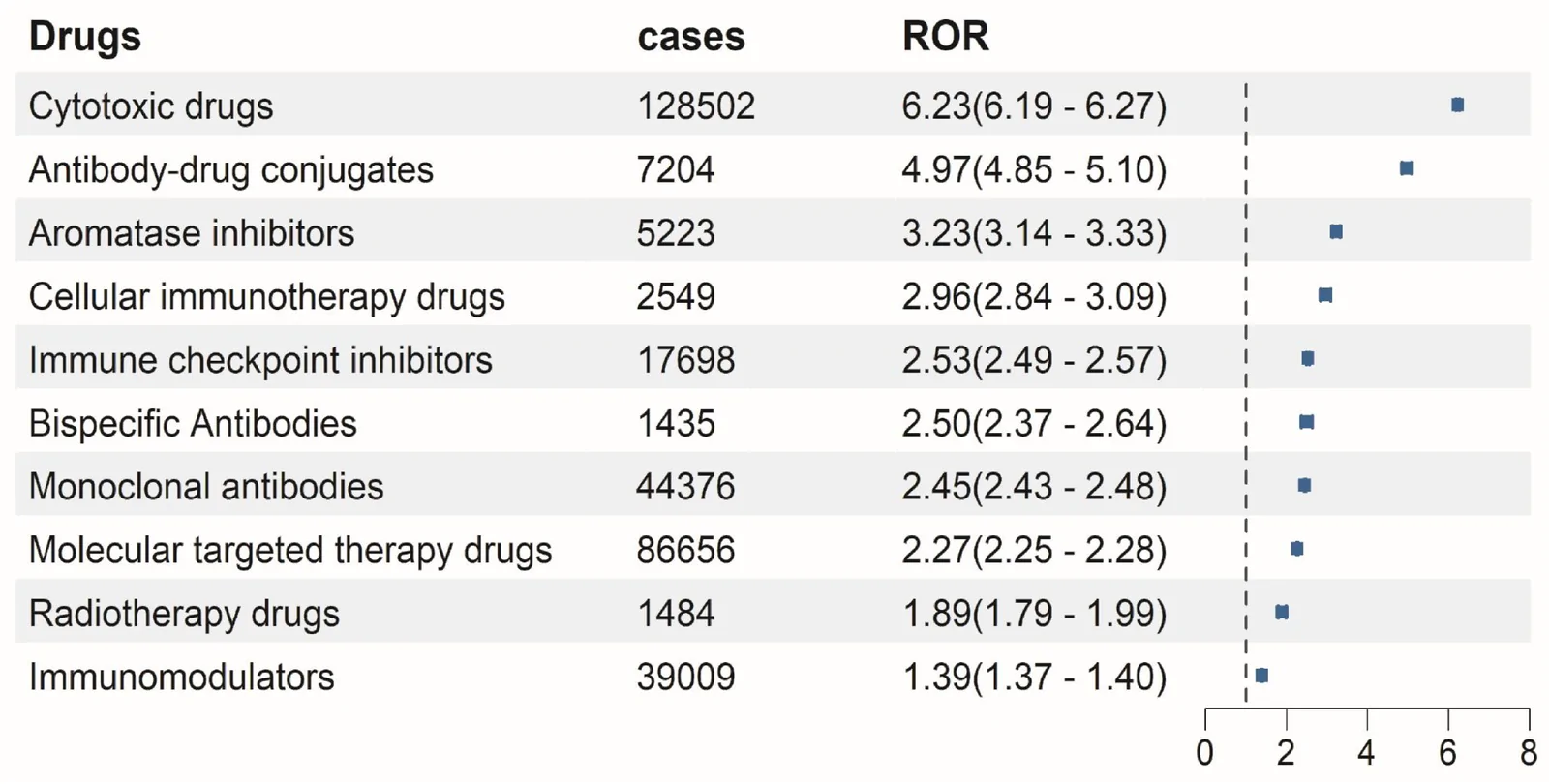
Forest plot of ROR values for haematotoxicity associated with various antineoplastic agents in the FAERS database.

### 3.3 Multivariable - adjusted signal detection for drug–event associations

Drugs suspected of being associated with an increased risk - defined as having more than 1, 000 case reports, a lower limit of the 95% confidence interval for the relative risk (ROR) greater than 5, and a p - adjusted value less than 0.01 - were selected for univariate analysis. Drugs with a p - value < 0.01 in the univariate analysis were subjected to LASSO regression, which identified 34 candidate drugs (**Figure 4**). These drugs, along with patient baseline characteristics, were then entered into a multivariate logistic regression model. The final multivariable logistic model demonstrated good discriminative ability (AUC = 0.62). After LASSO selection and Bonferroni correction, 27 antineoplastic agents showed statistically significant adjusted odds ratios (aOR) for reporting haematological toxicity compared with reports of other adverse events, including carboplatin, doxorubicin, gemcitabine, ranibizumab, cyclophosphamide, cytarabine, tislelizumab, and otolizumab, among others (**Figure 5**).

**Figure 4:**
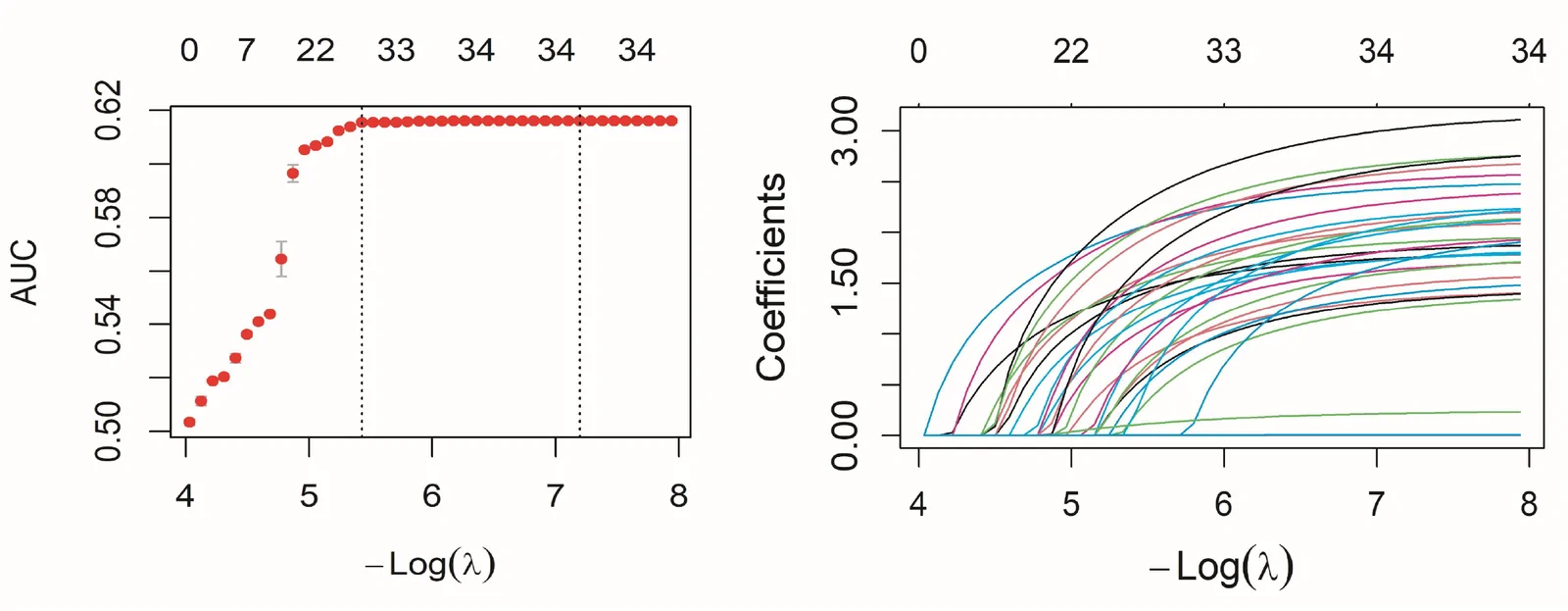
Results of LASSO regression analysis. LASSO, least absolute shrinkage and selection operator.

**Figure 5:**
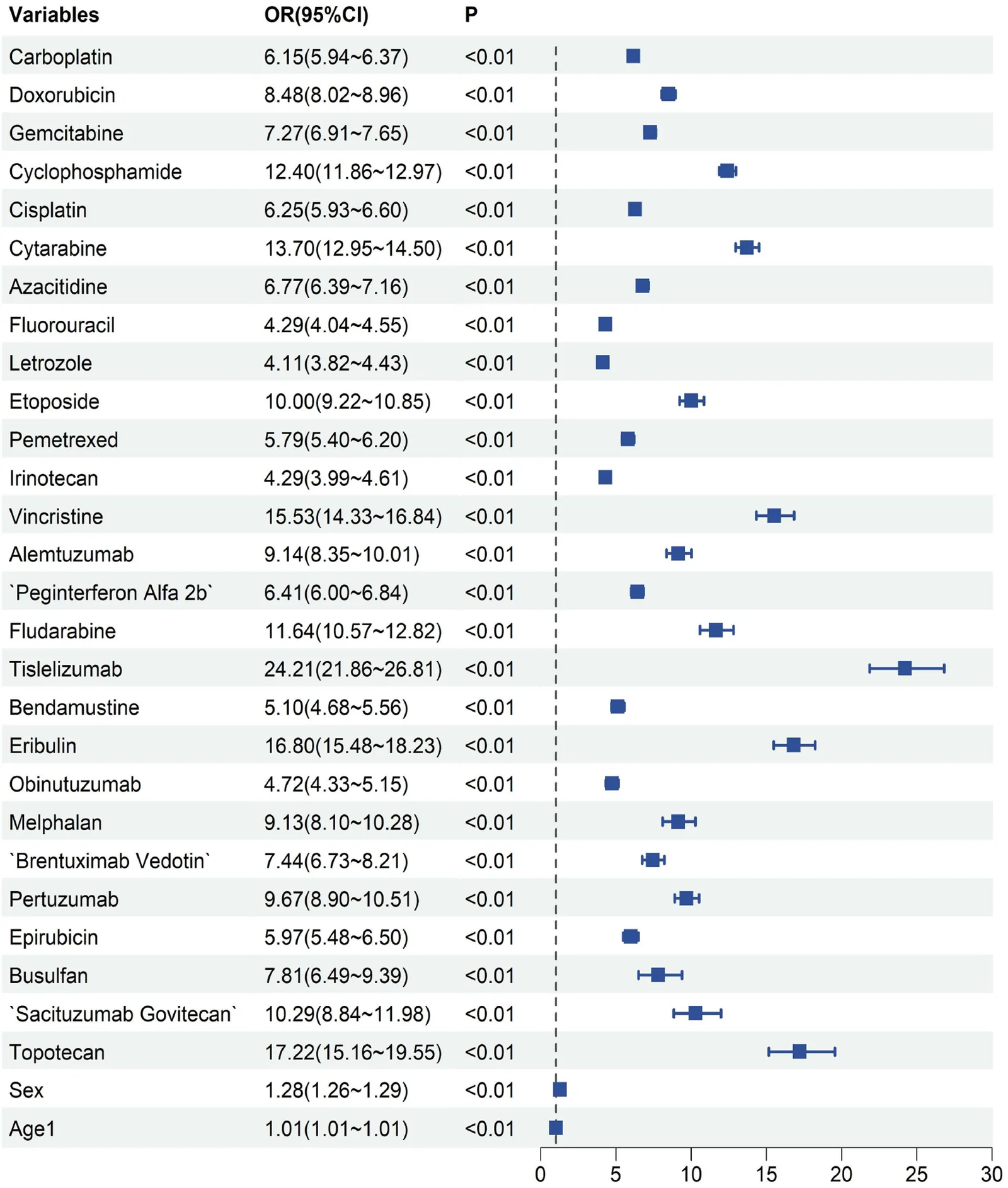
Results of multivariate logistic regression analysis. CI, confidence interval; OR, odds ratio; P - adjust, p - value after Bonferroni correction; p - adjust < 0.01, statistically significant.

### 3.4 Time to onset analysis

Systematic exclusion of inconsistent and missing data was performed to strengthen the reliability of TTO evaluation, which reduced the sample size of the final cohort relative to the original dataset. Inconsistent and missing date data were systematically excluded, included records with missing START_DT or EVENT_DT values, or where EVENT_DT preceded START_DT. This filtering process reduced the total number of reports in the evaluation cohort from 347, 248 to 147, 591 eligible reports (42.50% of the original cohort). A total of 1, 247 reports (0.36%) presented with TTO = 0 days and were assigned a TTO of 0.5 days for analysis. As shown in **Figure 6**, 61.65% of enrolled subjects (n = 90, 990) developed adverse events within 30 days post - treatment initiation. **Table 5** presents the TTO and WSP analytical outcomes for the top 30 anticancer drugs with haematological toxicity signals. According to the Weibull analysis, most of the top 30 drugs exhibited an early failure pattern, suggesting a high risk of haematological toxicity occurring early in the treatment course, with the risk gradually decreasing over time. To investigate potential selection bias, we compared the baseline characteristics of the TTO - evaluable cohort against the TTO - excluded cohort (**Supplementary Table S3**). The two groups were largely comparable: standardized mean differences (SMDs) were < 0.2 for age (SMD = 0.08), sex (SMD = 0.05), and drug class distribution (SMD range: 0.01 - 0.12). The Kaplan - Meier survival analysis illustrate the time to onset of haematological toxicity for anticancer drugs across different subgroups (**Figure 7**). The median time to onset was 21 days (IQR: 7 - 77) in male patients (n = 134, 495) and 21 days (IQR: 7 - 79) in female patients (n = 161, 501), with significant difference between the two groups (P < 0.0001). However, no significant difference was observed in the TTO of haematological toxicity between fatal and non - fatal cases (P = 0.4913); the median TTO was 21 days (IQR: 7 - 82) for fatal cases (n = 54, 840) and 21 days (IQR: 7 - 77) for non - fatal cases (n = 292, 408). Sensitivity analysis demonstrated that missing dates, reporting delays, and right - censoring did not exert any potential influence on the estimation of the Weibull shape parameter, yielding consistent results (**Supplementary Table S4**).

**Figure 6:**
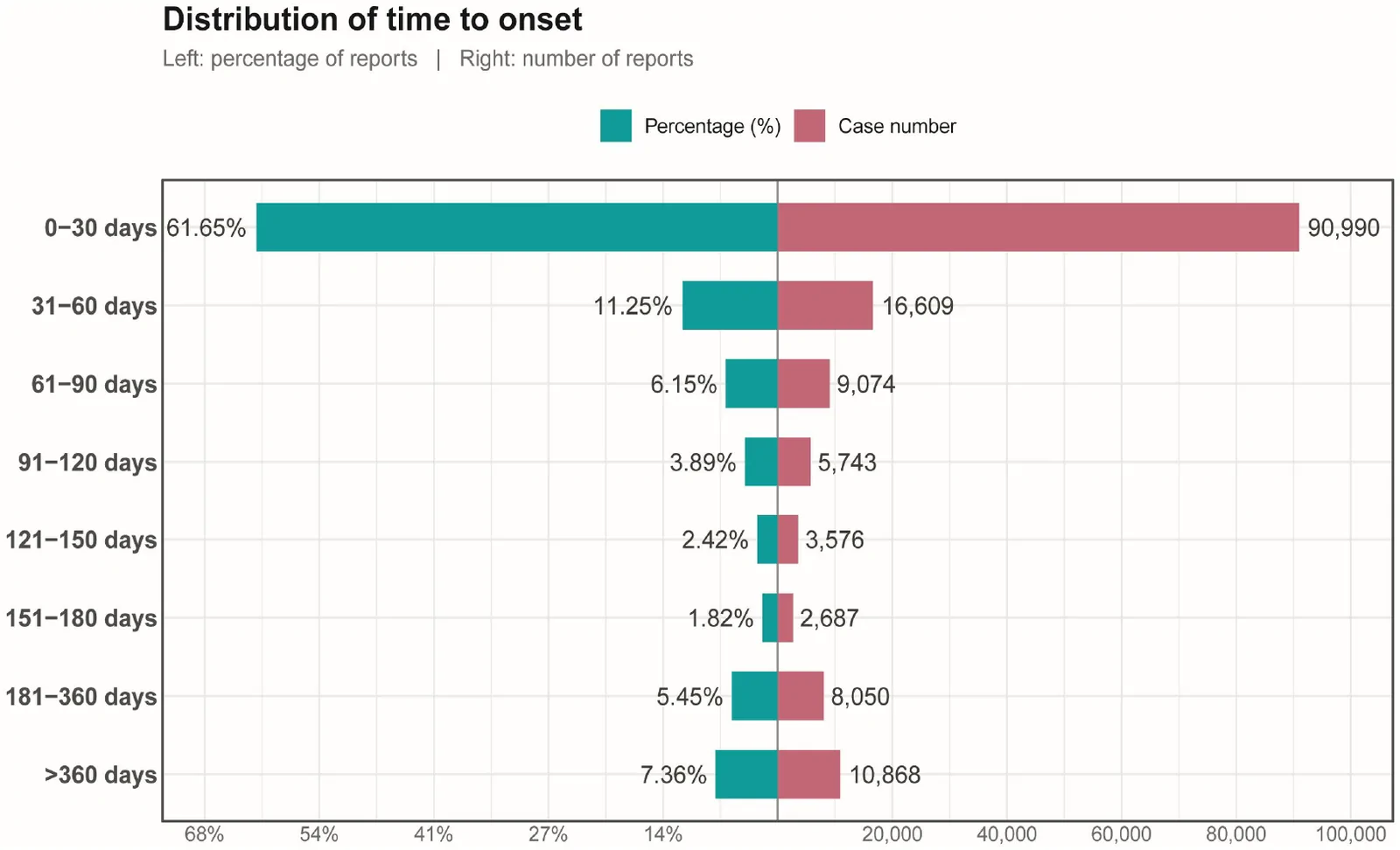
TTO of anti - neoplastic - associated haematotoxicity.

**Table 5:** TTO of haematotoxic adverse events across the top 30 antineoplastic agents.

SN	Drug	Cases	TTO (days)		Weibull distribution				Failure type
					Scale parameter		Shape parameter		
		n	Median	IQR	α	95% CI	β	95% CI	
1	Lenalidomide	7301	69	20 - 272	164.58	158.43 - 170.97	0.64	0.63 - 0.65	Early Failure
2	Carboplatin	4179	18	8 - 49	37.47	35.90 - 39.10	0.75	0.74 - 0.77	Random Failure
3	Rituximab	3966	31	10 - 119	82.76	78.24 - 87.55	0.59	0.57 - 0.60	Early Failure
4	Bevacizumab	3433	29	10 - 98	68.74	65.09 - 72.60	0.65	0.63 - 0.67	Early Failure
5	Cyclophosphamide	2744	10	7 - 23.25	27.96	26.16 - 29.89	0.60	0.58 - 0.61	Early Failure
6	Gemcitabine	2607	16	7 - 68.50	45.77	43.18 - 48.52	0.70	0.68 - 0.72	Early Failure
7	Paclitaxel	2569	14	7 - 50	37.87	35.72 - 40.14	0.71	0.69 - 0.73	Early Failure
8	Venetoclax	2492	17	7 - 51	41.98	39.32 - 44.82	0.64	0.62 - 0.66	Early Failure
9	Trastuzumab	2328	9	6 - 27	33.20	30.63 - 35.98	0.54	0.52 - 0.55	Early Failure
10	Docetaxel	2307	9	6 - 33	28.26	26.41 - 30.25	0.64	0.62 - 0.66	Early Failure
11	Ruxolitinib	2273	71	26 - 264	171.78	160.85 - 183.45	0.66	0.64 - 0.68	Early Failure
12	Nivolumab	2223	41	14 - 105	76.64	72.20 - 81.35	0.74	0.72 - 0.76	Early Failure
13	Methotrexate	2181	28	8 - 323	157.66	142.39 - 174.56	0.44	0.42 - 0.45	Early Failure
14	Oxaliplatin	2117	18	9 - 53	43.77	40.93 - 46.81	0.67	0.65 - 0.69	Early Failure
15	Pembrolizumab	2043	22	9 - 66.50	50.98	47.84 - 54.33	0.72	0.70 - 0.75	Early Failure
16	Azacitidine	1987	27	11 - 84	61.65	57.59 - 65.99	0.69	0.66 - 0.71	Early Failure
17	Doxorubicin	1868	16	8 - 57	42.63	39.70 - 45.79	0.68	0.66 - 0.70	Early Failure
18	Cytarabine	1845	11	7 - 20	22.00	20.66 - 23.43	0.77	0.75 - 0.79	Random Failure
19	Palbociclib	1779	39	16 - 191.50	129.48	119.51 - 140.29	0.62	0.60 - 0.64	Early Failure
20	Peginterferon Alfa 2A	1731	56	25 - 123	91.66	86.54 - 97.08	0.87	0.84 - 0.90	Random Failure
21	Letrozole	1600	39.5	17 - 220	138.14	126.79 - 150.51	0.61	0.59 - 0.63	Early Failure
22	Cisplatin	1584	14	8 - 35	29.22	27.38 - 31.19	0.80	0.78 - 0.83	Random Failure
23	Pomalidomide	1577	30	14 - 138	96.50	88.67 - 105.03	0.62	0.60 - 0.64	Early Failure
24	Capecitabine	1554	17	9 - 39	39.35	36.29 - 42.67	0.65	0.63 - 0.67	Early Failure
25	Bortezomib	1502	24	10 - 73	55.36	50.99 - 60.11	0.65	0.63 - 0.68	Early Failure
26	Fluorouracil	1480	18	10 - 52	41.74	38.74 - 44.97	0.73	0.70 - 0.75	Early Failure
27	Imatinib	1351	99	28 - 485	269.33	244.63 - 296.52	0.59	0.56 - 0.61	Early Failure
28	Sunitinib	1311	30	17 - 101	80.64	74.31 - 87.51	0.70	0.68 - 0.73	Early Failure
29	Ibrutinib	1157	92	23 - 366	213.73	193.21 - 236.43	0.60	0.58 - 0.63	Early Failure
30	Etoposide	780	12	8 - 32.25	29.73	26.64 - 33.19	0.68	0.65 - 0.71	Early Failure

**Figure 7:**
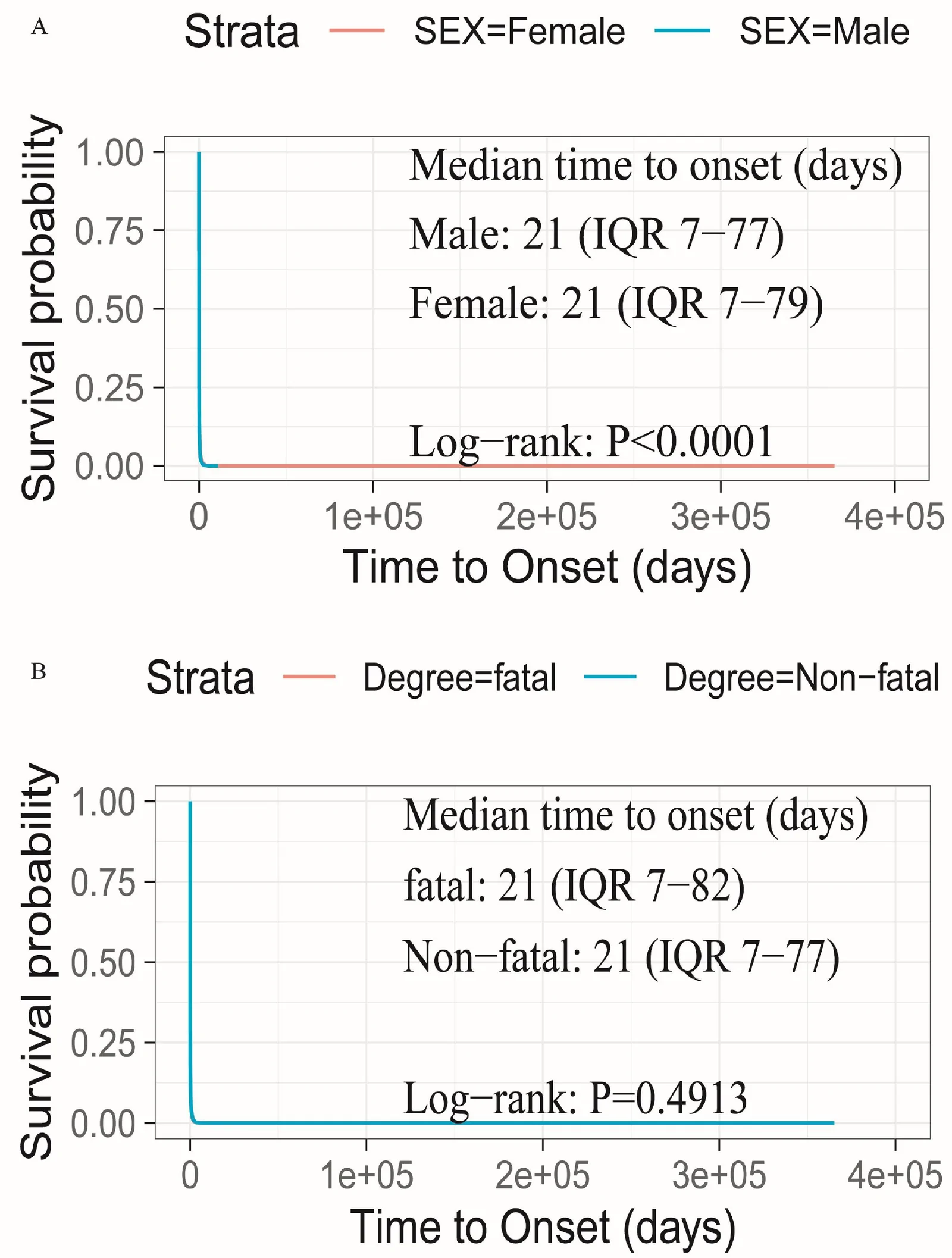
Kaplan - Meier curves for the TTO of antineoplastic - related haematological toxicity in different subgroups. **(A)** Sex stratification. **(B)** Fatal event status. Inter - group statistical significance was assessed via the Kaplan - Meier analysis with log - rank tests.

## 4 Discussion

Although haematological toxicity is a commonly encountered adverse reaction to anticancer treatment, large - scale studies examining its potential association with specific antineoplastic drugs are currently lacking. Accordingly, we extracted data from the FAERS database to identify drug entities that may be linked to this haematological complication. Cytotoxic drugs represented the most frequently reported drug category in case reports, with the United States and Japan contributing the largest number of reports. Demographic analyses revealed that the majority of adverse events occurred in female patients, and most AEs were observed in individuals aged 18 - 64.9 years, with a median age of 59 years. Regarding the onset timing, 61.65% of AEs developed within one month following treatment initiation. Importantly, life - threatening conditions or fatal outcomes were documented in 22.70% of the total cases.

This study found that female patients had a higher incidence of haematological toxicity than male patients when using anticancer drugs. Previous studies have shown that female cancer patients experience a higher proportion of treatment - related hematologic toxicity than men, and that this toxicity is also more severe. This may be related to differences in metabolic function between men and women, as these differences involve a variety of physiological and pathological processes (26). At the same time, women tend to have relatively lower glomerular filtration rates. Combined with potential differences in liver enzyme activity and drug transporter function between the sexes, these factors may collectively reduce drug clearance efficiency, ultimately leading to a significantly increased risk of adverse drug reactions (27). Although haematological adverse events from anticancer drugs occur most frequently in the 18 - 64.9 age group, older patients tend to develop more severe forms of such toxicity. This heightened vulnerability has been linked to progressive organ dysfunction, increased comorbidity rates, and reduced haematopoietic capacity associated with ageing (28). However, some real - world data reveal a different trend. An analysis based on the FAERS database showed that reports of drug - induced myelosuppression were primarily concentrated in the 18–65 age group (29). In studies of haematological toxicity associated with immune checkpoint inhibitors, patients aged 65 years or older actually had a lower risk compared with those under 65 (30). This discrepancy may reflect the prophylactic adjustment of starting doses for elderly patients in clinical practice.

Regarding the TTO of haematological toxicity, our analysis demonstrated that more than 60% of such cases emerged within the first month following treatment initiation. This finding confirms that most haematological toxicity events manifest during the early phase of treatment, which aligns with results reported in previous investigations (31, 32). A statistically significant difference in TTO was observed between male and female patients; however, no significant difference was found between fatal and non - fatal cases. This may be related to intrinsic sex differences in hematopoietic stem and progenitor cells. Studies suggest that androgen signalling pathways play an important role in the self - renewal and proliferation of hematopoietic stem cells (HSCs), and there may be inherent differences between men and women in the size and proliferation kinetics of the hematopoietic stem cell pool, which affect the speed at which the bone marrow responds to chemotherapy - induced damage (33, 34). Fatal clinical outcomes are predominantly governed by the severity and persistent duration of treatment - related toxicity, rather than the specific time point at which such toxicity first emerges. After the initiation of haematological toxicity, its early progression trajectory may present considerable interindividual consistency; nevertheless, subsequent downstream clinical outcomes are fundamentally determined by the host’ s baseline immune function, underlying disease progression status, pre - existing comorbidities, and the promptness of targeted supportive care interventions (35, 36). Taken together, these findings highlight the critical role of early recognition and timely management in preventing the exacerbation of antitumour drug - related haematological toxicity. The WSP test results indicate an early - failure pattern for most cases of drug - induced haematotoxicity, with events clustering in the early treatment phase. In light of this temporal pattern - which is consistent with the known biology of myelosuppression - clinicians should remain vigilant for early - onset haematological events during the initial phase of antineoplastic therapy. The finding supports the clinical prudence of routine blood count monitoring in early treatment cycles, a practice already recommended by standard oncology guidelines.

Further categorisation and analysis revealed that cytotoxic drugs had the highest ROR values and incidence rates. Cytarabine is a pyrimidine nucleoside analogue and belongs to the class of antimetabolites. It is primarily indicated for the induction and maintenance of remission in adults and children with acute non - lymphocytic leukaemia. It kills rapidly proliferating tumour cells by mimicking cytidine, a precursor required for cellular DNA synthesis, thereby inhibiting DNA polymerase and interfering with DNA replication. Studies have shown that haematological toxicity is the most common and dose - limiting adverse reaction associated with cytarabine treatment. At standard doses (100–200 mg/m², daily for 7 consecutive days), cytarabine induces haematological toxicity characterized by severe neutropenia, thrombocytopenia, and anaemia. These toxicities are intensified at higher doses and under combination treatment (37). Mechanistically, cytarabine non - selectively targets rapidly dividing bone marrow haematopoietic progenitor cells, causing substantial declines in circulating neutrophils, platelets, and red blood cells, thereby leading to life-threatening complications such as infection, haemorrhage, and anaemia (38). Notably, a growing body of evidence from clinical trials and translational studies has demonstrated that the magnitude of haematological toxicity associated with cytarabine may be modifiable through individualized dose de - escalation or refined dosing schedules (39, 40). Mechanistic studies have also documented that pharmacological inhibition of ABCC4 can enhance the sensitivity of leukaemic cells to cytarabine while potentially reducing off-target haematological toxicity (41). These observations from the clinical and translational literature suggest that further research is warranted to explore whether dose optimization strategies informed by pharmacogenomic or pharmacokinetic markers could mitigate cytarabine - associated myelosuppression without compromising anti - leukaemic efficacy. However, our FAERS - derived signal cannot and should not be used to recommend specific dose adjustments, as the database lacks the necessary pharmacokinetic, pharmacodynamic, and patient - level clinical data required to support such recommendations.

Multivariate analysis further indicated that cytotoxic drugs (carboplatin, doxorubicin, gemcitabine, cyclophosphamide, cisplatin, cytarabine, azacitidine, fluorouracil, etoposide, pemetrexed, irinotecan, vincristine, fludarabine, bendamustine, eribulin, melphalan, epirubicin, busulfan, topotecan), immune checkpoint inhibitors (tislelizumab), antibody - drug conjugates (gosatuzumab, vibutuximab), endocrine therapy agents (letrozole), and monoclonal antibodies (alencumab, obitumumab, patumumab) are all statistical signal for inducing drug - related haematological toxicity.

This study identified monoclonal antibodies as a prominent class of antitumour drugs linked to haematological toxicity, exemplified by alemtuzumab and obinutuzumab for chronic lymphocytic leukaemia (CLL) and other B - cell malignancies. Although these agents have revolutionised the treatment of diverse cancers, they are concurrently associated with a high rate of haematological side effects. Notably, as of July 2026, black - box warnings regarding haematological toxicity appeared in the product labels of six approved monoclonal antibodies (5.36%), and an additional 94 drugs (83.93%) included haematological toxicity warnings in their prescribing documentation (42). Existing literature consistently confirms that alemtuzumab confers a considerable risk of haematological toxicity, predominantly characterized by pancytopenia and delayed immune - mediated hematologic disorders (43). Mechanistically, alemtuzumab-mediated haematological toxicity results from global immune cell depletion. Following CD52 binding, alemtuzumab induces target cell death via ADCC and CDC. Since this activity lacks selectivity for malignant lymphocytes, it also extensively depletes normal lymphocytes, NK cells, and monocytes, leading to severe, sustained lymphocytopenia and subsequent immunosuppression (44). The literature reports multiple cases of obinutuzumab - induced acute thrombocytopenia, a rare but potentially life - threatening complication; potential mechanisms may include immune - mediated platelet destruction and impaired megakaryocyte maturation (45). In addition to the intrinsic toxicity of the drug, drug interactions can also exacerbate haematological toxicity. For example, the use of monoclonal antibodies in combination with chemotherapeutic agents significantly increases the risk of haematological toxicity (44). These findings highlight the need for strict monitoring of blood cell counts when monoclonal antibody drugs are used in clinical practice.

As a paradigm - shifting class of immunotherapies, immune checkpoint inhibitors (ICIs) enhance endogenous antitumour immunity by disrupting negative regulatory signals (e.g., PD - 1/PD - L1 and CTLA - 4), which restores T - cell activity against malignant cells. With proven sustained efficacy in diverse solid and haematological cancers, ICIs have fundamentally altered the management landscape for many oncological indications (45). Meta - analyses and real - world studies indicate that the anti - PD - 1 antibody tislelizumab confers a higher haematological toxicity risk than other ICIs. The most frequent adverse events with tislelizumab are haematological abnormalities, including anaemia, neutropenia, thrombocytopenia and leukopenia (46, 47). ICI - related haematological toxicity is uncommon but potentially life - threatening. It presents a wide spectrum from isolated cytopenia to pancytopenia, with complex pathogenesis, difficult diagnosis and limited standardized management (48). Haematological toxicity monitoring for ICI recipients requires both baseline assessment (via pre - treatment complete blood count) and ongoing surveillance throughout therapy. Because multiple factors can contribute to such toxicity, clinicians should rule out tumour infiltration of the bone marrow, infections (e.g., aspergillosis), drug interactions, and autoimmune diseases. For those on prolonged ICI regimens, early bone marrow aspiration biopsy is advisable to distinguish immune - mediated cytopenias from chemotherapy - induced suppression or uncommon entities like aplastic anaemia and myelofibrosis (49). Although the mechanisms underlying ICI - related haematological toxicity have not yet been fully elucidated, current research focuses on non - specific activation of the immune system, including excessive T - cell activation and the production of autoantibodies that attack red blood cells or other blood cell components (50).

Letrozole, a widely prescribed aromatase inhibitor for breast cancer, has a relatively mild myelosuppressive effect when used as monotherapy; however, its haematological toxicity warrants heightened vigilance in combination regimens or particular patient populations. For instance, concurrent use with CDK4/6 inhibitors is associated with a pronounced increase in haematological toxicity, especially neutropenia. Data from the PALOMA - 2 trial demonstrated that the palbociclib - letrozole combination was associated with a 95.30% incidence of all - grade neutropenia and a 67.10% rate of Grade 3/4 neutropenia, both significantly exceeding the corresponding rates in the placebo - letrozole arm. The pathogenesis of this haematological toxicity may be associated with patient - specific variability in therapeutic response. Despite its relatively high incidence, such toxicity is typically manageable via dose interruption, dose reduction, or conventional clinical interventions (51). These findings underscore the importance of routine blood count monitoring during letrozole therapy, enabling the prompt detection and management of haematological events - especially in elderly patients undergoing combination treatment.

Gosatuzumab is an antibody - drug conjugate that targets the Trop - 2 receptor; it is formed by conjugating a humanised anti - Trop - 2 monoclonal antibody to SN - 38 (the active metabolite of irinotecan) via a cleavable CL2A linker. SN - 38 is a topoisomerase I inhibitor. The main haematological toxicities associated with gosatuzumab are neutropenia and anaemia. Studies have shown that more than 5% of patients treated with gosatuzumab develop anaemia and febrile neutropenia, with a short median time to onset of just 12 days, suggesting that myelosuppression occurs rapidly. Haematological toxicity of this agent is mainly driven by non - specific payload release into the circulation. For sacituzumab govitecan, its cleavable linker may cause premature SN - 38 leakage into the bloodstream, where this topoisomerase I inhibitor damages rapidly dividing haematopoietic progenitors - including platelet, neutrophil, and erythroid lineages - in the bone marrow (52, 53). Consistent with previous reports, our analysis also implicated goseribulin as a contributor to haematological adverse events. This association is further supported by pharmacogenomic data showing that UGT1A1 polymorphisms are correlated with goseribulin - related toxicity in breast cancer patients (54). Furthermore, exposure - response analyses for goseribulin revealed a positive correlation between the incidence of all - grade neutropenia and peak plasma concentrations of total SN - 38. This finding implies that transiently high drug levels following administration may represent a critical driver of acute myelosuppression. Accordingly, a split - dose regimen (e.g., dosing on days 1 and 8) has been proposed to lower single - dose peak concentrations and thereby attenuate neutropenia severity (55). However, these hypotheses require prospective clinical evaluation, and our FAERS - based findings, which provide temporal signal confirmation rather than dose - response evidence and cannot be interpreted as supporting any specific dosing modification. The observed short median TTO for ADCs in our study should instead be viewed as a signal that warrants close pharmacovigilance monitoring in early treatment cycles.

This study is strengthened by a prolonged study timeframe, a large cohort, and rigorous data standardisation. To enhance the reliability of signal identification, we applied a multi - pronged analytical strategy encompassing ROR, PRR, and logistic regression, with the consistency of our results corroborated by subgroup and sensitivity analyses. However, several caveats warrant consideration. The FAERS system, being a spontaneous reporting database, is vulnerable to under - reporting and incomplete data - a particular concern for well recognised but non - severe haematological toxicities (e.g., mild anaemia), where reported frequencies likely fall below actual rates. Furthermore, the limited post - marketing experience with some novel antitumour agents constrains the comprehensiveness of their safety profiles. Notably, the frequent absence of dosage details, treatment schedules, and comorbidity data in individual reports hinders precise evaluation of haematological risk factors (56). Second, definitive causal relationships cannot be determined from FAERS analysis. This pharmacovigilance approach is designed to detect statistical signals rather than verify causality, which requires further clinical studies for confirmation. Reported associations between drugs and haematological toxicity may be influenced by underlying diseases, concomitant therapies or other confounding factors (57). The disproportionality analysis used herein only identifies statistical associations and does not establish a direct causal connection between anticancer drugs and the observed adverse events. A further limitation concerns the adjustment for potential confounders and the conduct of subgroup analyses. While the FAERS database permits stratification by age, sex, and selected factors, its utility for robust confounder correction is constrained by the absence or non - standardisation of many clinically relevant variables - such as liver and kidney function, genetic polymorphisms, and previous therapies (56). Thus, the associations detected in this study remain hypothesis - generating and require verification using rigorously designed epidemiological studies or clinical trial evidence.

### 4.1 Limitations

This study has several limitations that should be considered. Firstly, The multivariable logistic regression employed in this study was based on a case and non - case design within the FAERS spontaneous reporting database. Consequently, the adjusted odds ratios reflect the proportional increase in reporting odds for haematological toxicity relative to all other adverse events, rather than the absolute risk or cumulative incidence of haematological toxicity in exposed patients. Secondly, the high proportion of missing demographic data - particularly for body weight (65.40%) and age (27.97%) - represents an inherent limitation of the FAERS database. While we employed a missing - indicator approach and conducted sensitivity analyses to assess robustness, we cannot entirely exclude the possibility that unmeasured differences between the complete - case and missing - data subsets introduced residual confounding. Similarly, drug–drug co-prescriptions could not be fully accounted for, as FAERS captures only drugs reported at the time of the adverse event without reliable temporal sequencing or duration information. Finally, although we applied four complementary disproportionality algorithms, the inherent limitations of spontaneous reporting systems - including the absence of denominator data (total exposed population) and the potential for reporting bias - preclude the estimation of true incidence rates. The consistency of signals across multiple algorithms strengthens the robustness of our findings, but these results remain hypothesis-generating rather than confirmatory of causal associations. It is also important to note that all imbalance metrics derived from the FAERS database may be subject to various systemic biases, which could exaggerate or suppress the observed signals. For cytotoxic drugs, the net effect of high predictability combined with low novelty in reporting may underestimate the true risk, as clinicians may not report expected adverse events. For ICI and ADC therapies, the net effect of novelty and severity may overestimate the true risk, as clinicians are more likely to report unexpected and severe events. For combination therapy signals, the PS retention rule introduces signal sharing, in which both causative and non-causative factors may appear as positive signals.

## 5 Conclusion

This is the first pharmacovigilance study to systematically analyse anti - tumour drug - related haematological toxicity using the FAERS database. We identified 367 anti - tumour drugs with statistically significant reporting associations with haematological toxicity, of which 19 had no corresponding warnings in package inserts. The median TTO was 21 days in both fatal and non - fatal groups, with no significant difference. WSP analysis showed that 26 of the top 30 drugs followed the early failure model, supporting early - onset haematological toxicity. The primary scientific contribution of this work is to provide a comprehensive, class - stratified overview of haematological toxicity signals that can inform the design of future pharmacoepidemiological studies.

## Data Availability

The original contributions presented in the study are included in the article/**Supplementary Material**. Further inquiries can be directed to the corresponding author.
